# p73 is over-expressed in vulval cancer principally as the **Δ**2 isoform

**DOI:** 10.1054/bjoc.2001.2138

**Published:** 2001-11

**Authors:** J O'Nions, L A Brooks, A Sullivan, A Bell, B Dunne, M Rozycka, A Reddy, J A Tidy, D Evans, P J Farrell, A Evans, M Gasco, B Gusterson, T Crook

**Affiliations:** Ludwig Institute for Cancer Research, St Mary's Hospital Medical School, Norfolk Place, London, W2 1PG; University Department of Pathology, Glasgow University, Western Infirmary, Glasgow; Department of Molecular Medicine, King's College School of Medicine and Dentistry, Coldharbour Lane, London, SE5; Department of Gynaecological Oncology, University of Sheffield, Northern General Hospital, Sheffield, S5 7AU; Department of Histopathology, St Mary's Hospital Medical School, Norfolk Place, London, W2; Department of Surgery, Poole Hospitals NHS Trust, Poole, Dorset; UO Oncologia Medica, Azienda Ospedaliera S. Croce e Carle, Via Coppino 26, Cuneo, 12100, Italy

**Keywords:** vulval cancer, HPV, p73

## Abstract

p73 was studied in squamous cancers and precursor lesions of the vulva. Over-expression of p73 occurred commonly in both human papillomavirus (HPV)-positive and -negative squamous cell cancers (SCC) and high-grade premalignant lesions. Whereas expression in normal vulval epithelium was detected only in the basal and supra-basal layers, expression in neoplastic epithelium increased with grade of neoplasia, being maximal at both protein and RNA levels in SCC. p73 Δ2 was the principal over-expressed isoform in the majority of cases of vulval SCC and often the sole form expressed in SCC. Over-expression of p73 was associated with expression of HPV-encoded E7 or with hypermethylation or mutation of p16^INK4a^ in HPV-negative cases. There was a close correlation between expression of p73 and p14^ARF^ in cancers with loss of p53 function. The frequent over-expression of p73 Δ2 in neoplastic but not normal vulval epithelium, and its co-ordinate deregulation with other E2F-1 responsive genes suggests a role in the oncogenic process. © 2001 Cancer Research Campaign  http://www.bjcancer.com


British Journal of Cancer (2001) 85(10), 1551–1556
© 2001 Cancer Research Campaign
doi: 10.1054/ bjoc.2001.2138, available online at http://www.idealibrary.com on IDEAL

### p73 is over-expressed in vulval cancer principally as the Δ2 isoform

**J O'Nions[1], LA Brooks[1], A Sullivan[1], A Bell[2], B Dunne[2], M Rozycka[3], A Reddy[1], JA Tidy[4], D Evans[5], PJ Farrell[1], A Evans[6], M Gasco[7], B Gusterson[2] and T Crook[1]**

[1]Ludwig Institute for Cancer Research, St Mary's Hospital Medical School, Norfolk Place, London W2 1PG; [2]University Department of Pathology, Glasgow University, Western Infirmary, Glasgow; [3]Department of Molecular Medicine, King's College School of Medicine and Dentistry, Coldharbour Lane, London SE5; [4]Department of Gynaecological Oncology, University of Sheffield, Northern General Hospital, Sheffield, S5 7AU; [5]Department of Histopathology, St Mary's Hospital Medical School, Norfolk Place, London W2; [6]Department of Surgery, Poole Hospitals NHS Trust, Poole, Dorset; [7]UO Oncologia Medica, Azienda Ospedaliera S. Croce e Carle, Via Coppino 26, 12100, Cuneo, Italy

**Summary** p73 was studied in squamous cancers and precursor lesions of the vulva. Over-expression of p73 occurred commonly in both human papillomavirus (HPV)-positive and -negative squamous cell cancers (SCC) and high-grade premalignant lesions. Whereas expression in normal vulval epithelium was detected only in the basal and supra-basal layers, expression in neoplastic epithelium increased with grade of neoplasia, being maximal at both protein and RNA levels in SCC. p73 Δ2 was the principal over-expressed isoform in the majority of cases of vulval SCC and often the sole form expressed in SCC. Over-expression of p73 was associated with expression of HPV-encoded E7 or with hypermethylation or mutation of $p16^{INK4a}$ in HPV-negative cases. There was a close correlation between expression of p73 and $p14^{ARF}$ in cancers with loss of p53 function. The frequent over-expression of p73 Δ2 in neoplastic but not normal vulval epithelium, and its co-ordinate deregulation with other E2F-1 responsive genes suggests a role in the oncogenic process. © 2001 Cancer Research Campaign http://www.bjcancer.com

**Keywords**: vulval cancer; HPV; p73

Although vulval SCC is less common than cervical cancer, it is of major mechanistic interest since cancers are either HPV positive (principally HPV 16) or lack detectable HPV DNA sequences. HPV-positive cancers have an association with vulval intraepithelial neoplasia (VIN) (reviewed by Crum, 1992). Clear pathobiological differences between HPV-positive and HPV-negative cancers have been described (Crum, 1992). Allelotype analysis has revealed that no significant differences in sites of loss of heterozygosity (LOH) exist between the 2 forms of the disease (Pinto et al, 1999). However, a number of studies have revealed that mutation in p53 is more common in HPV-negative cancers (Lee et al, 1994; Marin et al, 2000; Brooks et al, 2001). Mechanistically, it is hypothesised that mutation in p53 functionally compensates for the absence of HPV 16E6, since this protein mediates inactivation of p53 via promotion of ubiquitin-dependent proteolysis. Despite the more common mutation of p53 in HPV-negative cases, a substantial number of vulval SCC occur which lack both mutation and HPV. The mechanism, if any, by which p53 function may be compromised in such cases is not known.

p73 has structural and functional homologies to p53, including sequence-specific DNA binding and transactivation functions (Kaghad et al, 1997). Over-expression of p73 is able to induce apoptosis in some human cancer lines (Jost et al, 1997) and expression of p73 has been shown to have a role in the differentiation of keratinocytes (De Laurenzi et al, 2000). p73 is expressed as a number of isoforms which arise by alternative splicing of exons encoding the –COOH terminus of the protein and which exhibit differences in trans-activating and growth suppressor functions (De Laurenzi et al, 1998; Ueda et al, 1999). The expression of a further spliced variant of p73 which lacks exon 2 (p73 Δ2) has been described in ovarian cancer (Ng et al, 2000) and in breast cancer cell lines (Fillippovich et al, 2001). Recently, p73 has been shown to be directly induced by E2F-1 and thereby to contribute to E2F-1-mediated apoptosis (Irwin et al, 2000; Lissy et al, 2000). Furthermore, both myc and adenovirus E1A can activate expression of p73 (Zaika et al, 2000).

Although p73 is subject to methylation-dependent transcriptional silencing in some B-cell malignancies, consistent with a role as a putative tumour-suppressor protein (Corn et al, 1999), mutational analyses have suggested that neither p63 nor p73 is frequently mutated in human cancers (Yoshikawa et al, 1999). Furthermore, p73 is over-expressed in some cancers, although the mechanism of this is unknown (Chi et al, 1999; Zaika et al, 1999). Assessment of the biological significance of the ability of p73 Δ2 to transdominantly inhibit both p53 and full-length p73 clearly requires analysis of the expression of this variant in a range of both normal and malignant tissues (Fillippovich et al, 2001). In the current study we have investigated the structure and expression of p73 in vulval neoplasia.

*Received 17 May 2001*
*Revised 23 July 2001*
*Accepted 7 August 2001*

*Correspondence to:* T Crook

### MATERIALS AND METHODS

### Tissues

SCC of the vulva and VIN III tissue samples were collected at operation. In each case the diagnosis was confirmed by routine histopathological analysis of resected tissue. Tissues were collected immediately into liquid nitrogen and stored until isolation of nucleic acids. Genomic DNA was isolated by proteinase K digestion and total RNA by RNAzol B. Cancers and VIN III were HPV-typed using standard PCR methodology. Paraffin sections of vulval neoplasia were retrieved from the Department of Histopathology at St Mary's Hospital, Paddington, London.

### Analysis of gene expression cDNA was synthesised with the ProStar system (Stratagene) from 3 µg of total RNA. Analysis of expression was performed by RT-PCR as described previously for p14$^{ARF}$ (Gazzeri et al, 1998), and p73 (De Laurenzi et al, 1998). For semi-quantitative analysis of gene expression, PCR was performed using the primers and thermal cycling conditions described and was for 22 cycles for p14$^{ARF}$, and 28 cycles for p73. In some experiments, amplification was extended to 40 cycles to analyse expression in tissues expressing a lower level of p73. Following PCR, reactions were resolved on agarose gels, transferred to Hybond-N$^{+}$ nylon and hybridised with $^{32}$P γ-ATP-labelled oligonucleotide probes specific for the amplified fragments. Analysis of N-terminal splice variants of p73 was performed using the primers described by Ng et al (2000). Identity of these was confirmed by cloning and sequencing and by hybridisation analysis of amplified products with oligonucleotide probes specific for exon 2 and exon 3 of p73. The presence of equivalent amounts of cDNA in each PCR was verified by amplification of β-actin under similar limiting conditions. RT-PCR analysis of p16$^{INK4a}$ was performed as described previously (Gonzalez-Zulueta et al, 1995).

### Immunocytochemistry

5 µm sections were cut from formalin-fixed, paraffin-embedded, tissue sections. The diagnosis in each case was confirmed by examination of haematoxylin and eosin-stained sections. For immunocytochemistry, sections were pressure-cooked in citrate buffer, then stained with antibodies: p14$^{ARF}$ goat polyclonal ((C20) Santa Cruz, SC-8613) was used at 1/100 dilution; p73 mouse monoclonal antibody Neomarkers, MS-764-P0, affinity-purified and diluted 1/150. Sections were scored independently by at least 2 pathologists. To demonstrate increase in p73 expression with increasing grade of neoplasia, sections were scored according to the following scheme. 1: basal staining only; 2: suprabasal staining where less than 50% of nuclei were positive in the strongest staining area in a high power field; 3: suprabasal staining where greater than 50% of nuclei were positive in the strongest stained area in a high-power field.

### Analysis of gene structure

Mutations in exon 1α and 2 of the INK4 locus were sought using single-strand conformation polymorphism analysis (SSCP) with primers and PCR conditions described by Zhang et al (1994). PCR reactions were resolved on 6% native acrylamide gels with and without 5% glycerol. Methylation of CpG sequences in the p16$^{INK4a}$ promoter was performed using methylation-specific PCR (MSP) as described by Herman et al (1996). To examine exon 1β for mutations, SSCP was performed using cDNA as the substrate and resolution on 6% gels as described above (Gazzeri et al, 1998). cDNA prepared from the Burkitt's lymphoma cell line Mutu was used as a positive control. Analysis of p73 coding sequences in the regions corresponding to the mutational hot spots of p53 was done by RT-PCR SSCP using conditions described by Kawano et al (1999).

### RESULTS

### Wild-type p73 is frequently over-expressed in vulval neoplasia

The expression of p73 was investigated in a series of vulval SCC and in VIN, previously analysed for the presence of HPV DNA sequences. In initial experiments, expression was analysed using RT-PCR methodology (De Laurenzi et al, 1998). This assay allows discrimination between alternatively spliced forms encoding different –COOH variants of p73 protein. Expression of p73 mRNA was detectable by RT-PCR in each of the 36 normal vulval epithelial samples available for analysis using 40 cycles of amplification. Expression was predominantly of α and γ variants in each of the normal vulval samples analysed. In cancers, expression was also restricted to the α and γ forms, but using limiting PCR conditions, was markedly increased relative to matched normal vulval epithelium in 29/36 cases examined (Figure 1). Interestingly, over-expression of p73 mRNA was detected in cancers both positive and negative for HPV DNA (Table 1A). To confirm that the elevated p73 mRNA levels were reflected in protein expression and to investigate possible associations between p73 expression and grade of neoplasia, we performed immunocytochemical analysis of tissue sections of VIN and SCC cut from paraffin blocks. These studies revealed that expression of p73 was detectable in each case of normal epithelium, consistent with RT-PCR analysis performed under extended cycling conditions (Figure 2). Expression was restricted to the basal and supra-basal layers in normal tissue, but this restriction was lost with increasing grade (Figure 2). To further demonstrate the increase in expression with grade of neoplasia, expression was scored using a semi-quantitative immunocytochemical technique in VIN. A mean expression index was calculated for each of VIN I, VIN II and VIN III. This clearly demonstrated the increase in expression from VIN I to VIN III (Table 1B). The presence of mutations in the over-expressed p73 mRNA was sought using RT-SSCP (Kawano et al, 1999). No mobility shifts suggestive of mutation were detected in 24 vulval SCC analysed.

### p73 Δ2 is the predominant over-expressed form of p73

The high frequency of over-expression of p73α and γ in vulval SCC, in the absence of mutations, was unexpected in view of the pro-apoptotic and negative growth-regulatory functions of these proteins. We therefore performed additional RT-PCR analysis to examine the N-termini of the mRNA species. There was over-expression of p73 Δ2 in the majority of vulval SCC shown to over-express p73α (Figure 1 and Table 1A). Full-length p73 was only rarely simultaneously over-expressed, p73 Δ2 being the only form expressed in the majority of cases (Figure 1 and Table 1A).

### Inactivation of p16$^{INK4a}$ correlates inversely with the presence of HPV

The ability of HPV E7 to deregulate p73 expression via E2F-1 and pRb provides a mechanistic explanation for over-expression

 *© 2001 Cancer Research Campaign*

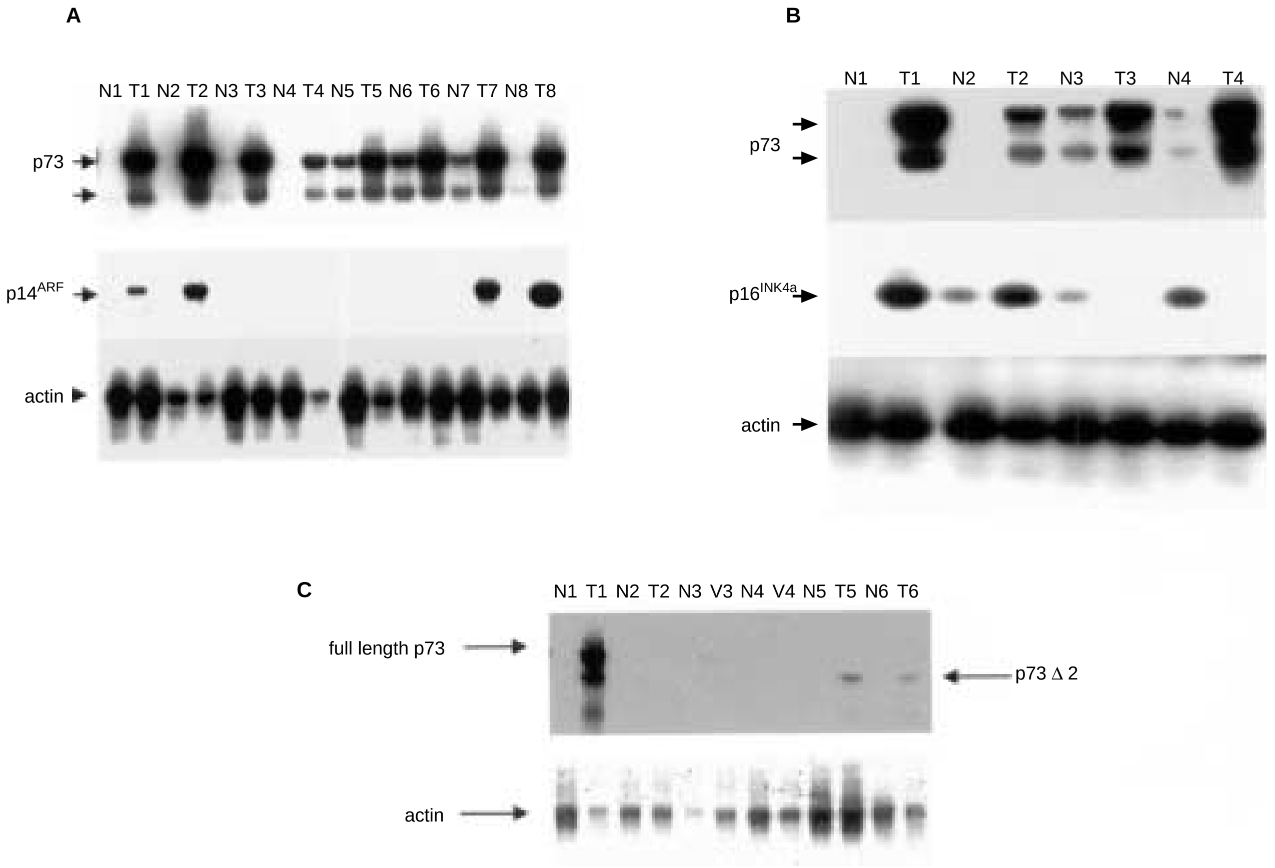

**Figure 1** (**A**) p73 expression in vulval neoplasia. RT-PCR analysis of p73 expression in paired normal (N) and tumour (T). PCR was performed for 28 cycles. p73 is predominantly expressed as the $\alpha$ and $\gamma$ isoforms (upper and lower arrows respectively). The middle panel shows that expression of $p14^{ARF}$ is deregulated with p73 in a subset of vulval SCC. Cancers T3, T4, T5 and T6 which do not have deregulated $p14^{ARF}$ lack HPV DNA and mutant p53. The lower panel is actin. (**B**) Expression of p73 in vulval neoplasia can be deregulated in the presence of either HPV DNA or transcriptional silencing of $p16^{INK4a}$. T1 and T2 (each with matched normal N1 and N2) are HPV positive vulval SCC and over-express $p16^{INK4a}$ mRNA. T3 and T4 (also with matched normal) are vulval SCC with hypermethylated $p16^{INK4a}$ sequences and concomitant absence of p16 transcript. p73 RNA is over-expressed in both groups of cancer. PCR was performed for 28 cycles for both p73 and $p16^{INK4a}$. (**C**) Vulval cancers over-express p73 $\Delta$2. T1 is a vulval SCC which over-expresses both full-length and $\Delta$2 forms, whereas T5 and T6 express only the $\Delta$2 form. V3 and V4 are VIN I and do not express detectable p73 RNA under these limiting PCR conditions (28 cycles of PCR). Note also the absence of detectable p73mRNA in each matched normal (N1–N6) under these conditions

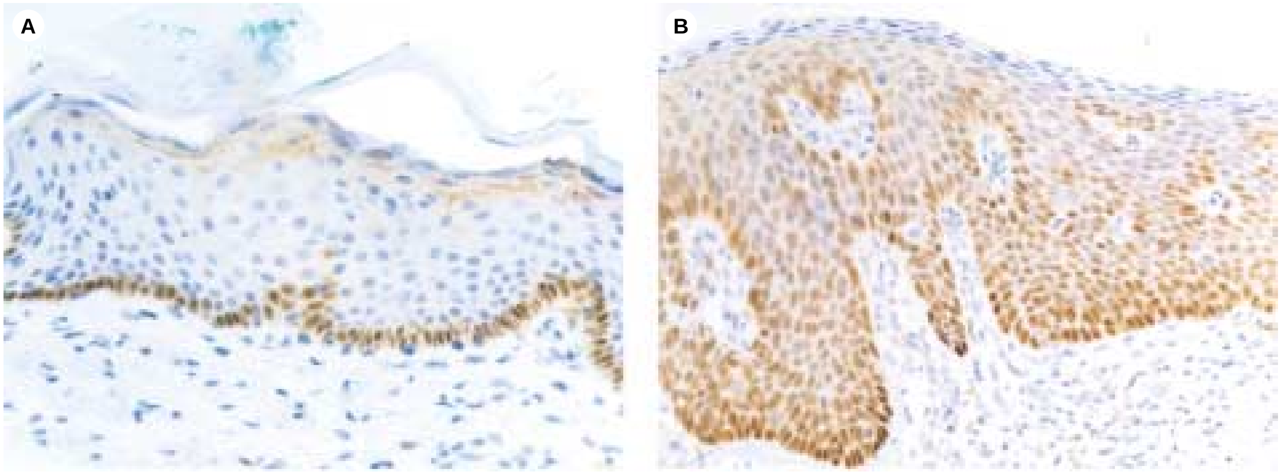

**Figure 2** Immunocytochemical analysis of expression of p73 in normal and neoplastic vulval epithelium. Sections were prepared from formalin-fixed, parrafin-embedded tissue samples as described in Materials and Methods.(**A**) Expression of p73 in normal vulval epithelium is in the basal and immediately supra-basal cells. RT-PCR showed that this was predominantly p73$\alpha$ and p73$\gamma$. (**B**) Expression of p73 in VIN II

of p73 in HPV-positive cancers. We were interested to identify alternative mechanisms by which pRb function might be inhibited, to determine how p73 was deregulated in cases lacking HPV. To address this issue, we analysed the structure and expression of $p16^{INK4a}$ to determine whether mutation and/or epigenetic silencing of the gene was related to deregulation of p73 in

© 2001 Cancer Research Campaign

**Table 1A** Expression of p14, p16 and p73 in vulval cancers

| Cancer | p53 status | HPV | p16$^{INK4a}$ RNA | p16$^{INK4a}$ meth. | FL p73 | p73Δ2 | p14$^{ARF}$ |
|---|---|---|---|---|---|---|---|
| 1 | Mt | – | – | Yes | – | + | + |
| 2 | Mt | – | – | Yes | – | + | + |
| 3 | Mt | – | – | Yes | + | + | + |
| 4 | Mt | – | – | – | + | + | + |
| 5 | Mt | – | – | – | – | + | + |
| 6 | Mt | – | – | Yes | – | + | – |
| 7 | Mt | – | – | Yes | – | + | – |
| 8 | Mt | – | + | – | – | – | – |
| 9 | Mt | – | – | Yes | – | + | + |
| 10 | Mt | – | – | Yes | – | – | + |
| 11 | Mt | – | – | – | – | – | – |
| 12 | Mt | 16 | + | – | + | + | + |
| 13 | Mt | – | – | Yes | – | – | + |
| 14 | Mt | 16 | + | – | + | + | + |
| 15 | Mt | – | – | – | – | – | + |
| 16 | Mt | – | – | – | – | – | + |
| 17 | Mt | – | Mt[a] | – | – | + | + |
| 18 | Mt | – | – | Yes | – | + | – |
| 19 | Mt | – | + | – | – | + | + |
| 20 | Mt | 16 | + | – | – | + | + |
| 21 | Mt | 16 | + | – | – | + | + |
| 22 | Wt | 16 | + | – | – | + | + |
| 23 | Wt | – | – | – | + | + | + |
| 24 | Wt | 16 | + | – | – | + | + |
| 25 | Wt | 16 | + | – | – | + | + |
| 26 | Wt | – | + | – | – | – | – |
| 27 | Wt | 16 | + | – | + | + | + |
| 28 | Wt | 16 | + | – | – | + | + |
| 29 | Wt | 16 | + | – | – | + | + |
| 30 | Wt | – | – | Yes | – | + | – |
| 31 | Wt | – | – | Yes | – | + | – |
| 32 | Wt | 16 | + | – | – | + | + |
| 33 | Wt | – | Mt[b] | – | – | + | – |
| 34 | Wt | 16 | + | Yes | – | + | – |
| 35 | Wt | – | Mt[c] | – | – | + | – |
| 36 | Wt | 16 | – | Yes | – | + | + |

Expression was determined by RT-PCR as described in Methods and Materials. Mt = mutant, Wt = wild-type. FL p73 = full-length p73. Δ2 p73 = deleted exon 2 p73. + denotes expression of p73 and p14$^{ARF}$ increased relative to matched normal tissue. + denotes detectable expression of p16$^{INK4a}$. The mutations in p16$^{INK4a}$ are: [a] codon 80 Cga >TgA = Arg >Ter; [b] codon 11 CCT > CTT = Pro > Leu; [c] codon 48 CCg >TCg = Pro > Ser. The polymorphism at codon 148 Ala > Thr was detected in 4/36 individuals analysed.

**Table 1B** Effect of grade of neoplasia on expression of p73

| | VIN I | VIN II | VIN III | SCC |
|---|---|---|---|---|
| Mean score | 1.2 +/– 0.46 | 1.42+/– 0.37 | 2.25 +/–0.68 | 2.59+/–0.44 |

Data shown are staining indices, determined as described in Methods, +/– standard deviation.

HPV negative cases. Using MSP, the presence of hypermethylation in the p16$^{INK4a}$ gene promoter was detected in 13/36 cases of vulval SCC (Figure 3, Table 1A). Mutations in p16$^{INK4a}$ were detected in 3/36 vulval SCC (Figure 4), all 3 cases being HPV-negative. No mutations were detected in p16$^{INK4a}$ in 21 HPV16-positive vulval SCC or in 78 HPV16-positive cervical SCC. Taken together, of the 16 cases of vulval SCC with inactivation of p16$^{INK4a}$, only one was HPV16-positive. Over-expression of p73 occurred in 15/16 cases of HPV-negative vulval SCC with p16$^{INK4a}$ inactivation (Table 1A, Figure 1). These data imply that inactivation of p16$^{INK4a}$ and expression of HPV16 E7 may be functionally interchangeable events in vulval neoplasia and that either can result in p73 over-expression.

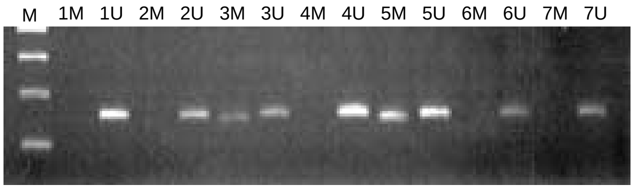

**Figure 3** MSP analysis of CpG methylation in the p16$^{INK4a}$ gene promoter in vulval neoplasia. Each paired lane represents either methylated (M) or unmethylated (U) DNA for each vulval SCC. Note the presence of methylated DNA in cancers 3 and 5. The presence of unmethylated DNA in both cases is attributable either to the presence of normal tissue within the tumour biopsy or hemimethylation of the CpG sequences. M = DNA molecular weight markers

 *© 2001 Cancer Research Campaign*

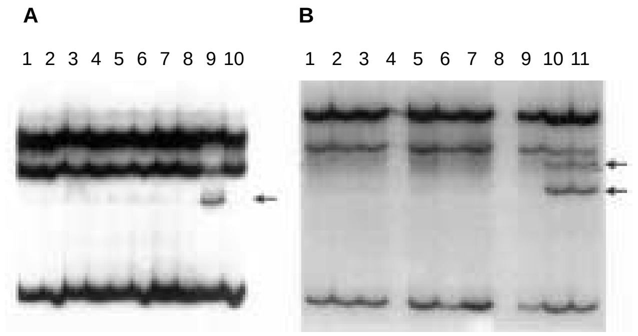

**Figure 4** Mutations occur in exon 2 of p16$^{INK4a}$ in vulval but not cervical SCC. The autoradiographs show SSCP analysis of exon 2 of INK4 in anogenital neoplasia. (**A**) 5′ portion of exon 2. Lanes 1–8 are cervical SCC. Lane 9 is vulval cancer with mutation. (**B**) 3′ portion of exon 2. Lanes 1–9 are cervical SCC. Lanes 10 and 11 are vulval SCC, each with polymorphism at codon 148 of p16$^{INK4a}$. The abnormal mobility bands are indicated by arrows

### p73 expression is deregulated with p14$^{ARF}$ in cancers with loss of p53 function

Because p73 is deregulated by E2F-1 expression (Irwin et al, 2000; Lissy et al, 2000), we determined the expression levels of another recognised E2F-1 regulated gene, p14$^{ARF}$ (Bates et al, 1998), in cancers with p73 over-expression. Using RT-PCR, we determined whether p73 and p14$^{ARF}$ were over-expressed together in vulval cancers either positive for HPV, mutant for p53 or having neither (Figure 1). 29/36 vulval SCC analysed by RT-PCR over-expressed p73 and there was concomittant over-expression of p14$^{ARF}$ in 21/29. Of these, 20 cases were either mutant for p53 or positive for HPV 16. In total, 6 vulval SCC were both negative for HPV and contained wild-type p53 sequence. Of these 6 cases, p73 was over-expressed in 5, but p14$^{ARF}$ in only a single case. These results suggest that deregulation of p14$^{ARF}$, but not p73, requires loss of p53 function.

### DISCUSSION

In this work we show that expression of p73 is deregulated at both protein and mRNA levels in a high proportion of vulval carcinomas and pre-malignant lesions. Our results are consistent with studies which have reported over-expression of p73 in other common cancers, including breast (Zaika et al, 1998), bladder (Chi et al, 1999) and hepatocellular carcinoma (Tannapfel et al, 1999). We also make the important observation that deregulation is frequently of the Δ2 transdominant form of p73.

p73 exists as a number of isoforms, generated by alternative splicing of exons encoding the -COOH terminus of the protein (De Laurenzi et al, 1998; Ueda et al, 1999). Multiple isoforms are expressed in keratinocytes (De Laurenzi et al, 1998). In cervical epithelium the α form is the overwhelmingly predominant variant expressed (data not shown). It is of interest, therefore, that p73 expression is predominantly of the α and γ forms in vulval epithelium. It is also noteworthy that whereas expression of p73 is restricted to the basal and supra-basal layers in normal epithelium, this restriction is lost in VIN and in SCC, expression frequently encompassing the entire epithelium. A further observation of interest was the increase in expression with increasing grade of neoplasia. Over-expression of p73 protein has been correlated with progression of other cancers, for example in the bladder, where p73-positive cancers are associated with poor prognosis (Tannapfel et al, 1999).

In view of the hypothesised role of p73 as a tumour-suppressor protein, it was perhaps surprising to observe over-expression in such a high proportion of cases. Sequence analysis did not detect mutations in p73 in vulval cancers, findings consistent with other authors' studies of both solid and haematological malignancies (Yoshikawa et al, 1999). A variant of p73 which excludes exon 2 (p73 Δ2) was recently described in ovarian carcinomas (Ng et al, 2000) and in some breast cancer cell lines (Filippovich et al, 2001). The authors of the latter study stressed the importance of analysing expression of the Δ2 form in a range of normal and malignant tissues. In the present study, we make the interesting observation that, in vulval cancer, p73 over-expression is indeed predominantly of the Δ2 form, and in some cases in our series this was the only form of p73 detectable. p73 Δ2 has been shown to inhibit the transactivating function of both p53 and full-length p73 (Filippovich et al, 2001). As such, the over-expression of p73 Δ2 forms suggests a contribution to tumourigenesis in vulval SCC by transdominant inhibition of wild-type p53 and full-length p73. It is also an interesting possibility that over-expression of p73Δ2 may be related to the differentiation status of squamous cancers. The importance of full-length p73 expression in mediating keratinocyte differentiation has been clearly demonstrated (De Laurenzi et al, 2000). It is, therefore, an attractive hypothesis that impaired differentiation of some squamous cancers may, at least in part, result from transdominant inhibition of full-length p73-dependent differentiation by the Δ2 variant. Additional studies to address this hypothesis would clearly be of interest.

Expression of p73 is driven by E2F-1 (Irwin et al, 2000; Lissy et al, 2000). It was therefore of interest to investigate potential mechanisms by which E2F-1 is itself deregulated in vulval cancer. A subset of the SCC contained and expressed HPV 16 DNA sequences. HPV16 E7 associates with pRb and thereby deregulates expression of E2F-1 responsive genes such as B-*myb* (Lam et al, 1994), providing a mechanistic explanation for p73 deregulation in HPV-positive cases. Analysis of HPV-negative cases revealed frequent abnormalities in p16$^{INK4a}$, either in the form of point mutations or methylation-dependent transcriptional silencing. Inactivation of p16$^{INK4a}$ was inversely correlated with the presence of HPV DNA in the majority of cases, implying that loss of p16$^{INK4a}$ function can, at least partially, compensate for the absence of HPV E7 expression. Support for this hypothesis is afforded by the consistent absence of p16$^{INK4a}$ mutations in a large series of HPV-positive cervical SCC, whereas mutations in p16$^{INK4a}$ were detected in 3/16 HPV-negative vulval SCC.

Support for the hypothesis that over-expression of p73 results from E2F-1 deregulation was provided by the observation of co-ordinate over-expression with p14$^{ARF}$ in a large proportion of vulval cancers with simultaneous loss of p53 function (via mutation or HPV 16 positivity). Interestingly, vulval cancers lacking a p53 mutation and HPV 16 did not, in general, over-express p14$^{ARF}$ despite frequent, abundant over-expression of p73. Previous studies have revealed that cells over-expressing p14$^{ARF}$ are almost always null for p53 (Stott et al, 1998). Our data are consistent with this hypothesis and also suggest that deregulation of p73 is not dependent on the p53 status of the cell.

In conclusion, our results add to the growing evidence that p73 over-expression is a common event in human neoplasia and provide direct support from clinical biopsy material for E2F 1-driven p73 deregulation in vivo.

© 2001 Cancer Research Campaign

### ACKNOWLEDGEMENTS

Research in the laboratory of B Gusterson is supported by Breakthrough Breast Cancer. We are most grateful to Dr W Kaelin for providing the monoclonal antibody to p73.

### REFERENCES

Bates S, Phillips AC, Clarke PA, Stott F, Peters G, Ludwig RL and Vousden KH (1998) p14ARF links the tumour suppressors RB and p53. *Nature* **395**: 124–125

Brooks LA, Tidy JA, Gusterson B, Hiller L, O'Nions J, Gasco M, Marin MC, Farrell PJ, Kaelin WG Jr and Crook T (2000) Preferential retention of codon 72 arginine p53 in squamous cell carcinomas of the vulva occurs in cancers positive and negative for human papillomavirus. *Cancer Res* **60**: 6875–6877

Chi S-G, Chang S-G, Lee S-J, Lee C-H, Kim JI and Park J-H (1999) Elevated and biallelic expression of p73 is associated with progression of human bladder cancer. *Cancer Res* **59**: 2791–2793

Corn PG, Kuerbitz SJ, van Noesel MM, Esteller M, Compitello N, Baylin SB and Herman JG (1999) Transcriptional silencing of the p73 gene in acute lymphoblastic leukemia and Burkitt's lymphoma is associated with 5′CpG methylation. *Cancer Res* **59**: 3352–3356

Crum CP (1992) Carcinoma of the vulva: epidemiology and pathogenesis. *Obstet Gynecol* **79**: 448–454

De Laurenzi V, Costanzo A, Barcaroli D, Terrinoni A, Falco M, Annicchiarico-Petruzzelli M, Levrero M and Melino G (1998) Two new splice variants, gamma and delta, with different transcriptional activity. *J Exp Med* **188**: 1763–1768

De Laurenzi V, Rossi A, Terrinoni A, Barcoroli D, Levrero M, Costanzo A, Knight RA, Guerrieri P and Melino G (2000) p63 and p73 transactivate differentiation gene promoters in human keratinocytes. *Biochem Biophys Res Com* **273**: 342–346

Fillipovich I, Sorokina N, Gatei M, Haupt Y, Hobson K, Moallem E, Spring K, Mould M, McGuckin MA, Lavin MF and Khanna KK (2001) Transactivation-deficient p73alpha (p73 Delta exon2) inhibits apoptosis and competes with p53. *Oncogene* **20**: 514–522

Gazzeri S, Della Valle V, Chaussade L, Brambilla C, Larsen CJ and Brambilla E (1998) The human p19ARF protein encoded by the beta transcript of the p16INK4a gene is frequently lost in small cell lung cancer. *Cancer Res* **58**: 3926–3931

Gonzalez-Zulueta M, Bender CM, Yang AS, Nguyen T, Beart RW, Van Tornout JM and Jones PA (1995) Methylation of the 5′CpG island of the p16/CDKN2 tumor suppressor gene in normal and transformed human tissues correlates with gene silencing. *Cancer Res* **55**: 4531–4535

Herman JG, Graff JR, Myohanen S, Nelkin BD and Baylin SB (1996) Methylation-specific PCR: a novel assay for methylation status of CpG islands. *Proc Natl Acad Sci USA* **93**: 9821–9826

Irwin M, Marin MC, Phillips AC, Seelan RS, Smith DI, Liu W, Flores ER, Tsai KY, Jacks T, Vousden KH and Kaelin WG Jr (2000) Role for the p53 homologue p73 in E2F-1-induced apoptosis. *Nature* **407**: 645–648

Jost CA, Marin MC and Kaelin WG Jr (1997) p73 is a simian p53-related protein that can induce apoptosis. *Nature* **389**: 191–194

Kaghad M, Bonnet H, Yang A, Creancier L, Biscan JC, Valent A, Minty A, Chalon P, Lelias JM, DuMont X, Ferrara P, McKeon F and Caput D (1997) Monoallelically expressed gene related to p53 at 1p36, a region frequently deleted in neuroblastoma and other human cancers. *Cell* **90**: 809–819

Kawano S, Miller CW, Gombart AF, Bartram CR, Matsuo Y, Hiroya A, Akiko S, Said J, Tatsumi E and Koeffler HP (1999) Loss of p73 gene expression in leukemias/lymphomas due to hypermethylation. *Blood* **94**: 1113–1120

Lam EW, Morris JD, Davies R, Crook T, Watson RJ and Vousden KH (1994) HPV16 E7 oncoprotein deregulates B-*myb* expression: correlation with p107/E2F complexes. *EMBO J* **13**: 871–878

Lee YY, Wilcznski SP, Chumakov A, Chih D and Koeffler HP (1994) Carcinoma of the vulva: HPV and p53 mutations. *Oncogene* **9**: 1655–1659

Lissy NA, Davis PK, Irwin M, Kaelin WG and Dowdy SF (2000) A common E2F-1 and p73 pathway mediates cell death induced by TCR activation. *Nature* **407**: 642–645

Marin MC, Jost CA, Brooks LA, Irwin MS, O'Nions J, Tidy JA, James N, McGregor JM, Harwood CA, Yulug IG, Vousden KH, Allday MJ, Gusterson B, Ikawa S, Hinds PW, Crook T and Kaelin WG Jr (2000) A common polymorphism acts as an intragenic modifier of mutant p53 behaviour. *Nat Gen* **25**: 47–54

Ng S-W, Yiu GK, Liu Y, Huang L-W, Palnati M, Jun SH, Berkowitz RS and Mok SC (2000) Analysis of p73 in human borderline and invasive ovarian tumors. *Oncogene* **19**: 1885–1890

Pinto AP, Lin M-C, Mutter GL, Sun D, Villa LV and Crum CP (1999) Allelic loss in human papillomavirus-positive and -negative vulvar squamous cell carcinomas. *Am J Path* **154**: 1009–1015

Stott FJ, Bates S, James MC, McConnell BB, Starborg M, Brookes S, Palmero I, Ryan K, Hara E, Vousden KH and Peters G (1998) The alternative product from the human CDKN2A locus, p14 (ARF), participates in a regulatory feedback loop with p53 and MDM2. *EMBO J* **17**: 5001–5014

Tannapfel A, Wasner M, Krause K, Geissler F, Katalinic A, Hauss J, Mossner J, Engeland K and Wittekind C (1999) Expression of p73 and its relation to histopathology and prognosis in hepatocellular carcinoma *J Natl Cancer Inst* **91**: 1154–1158

Ueda Y, Hijikata M, Takagi S, Chiba T and Shimotohno K (1999) New p73 variants with altered C-terminal structures have varied transcriptional activities. *Oncogene* **18**: 4993–4998

Yoshikawa H, Nagashima M, Khan MA, McMenamin MG, Hagiwara K and Harris CC (1999) Mutational analysis of p73 and p53 in human cancer cell lines. *Oncogene* **18**: 3415–3421

Zaika A, Irwin M, Sansome C and Moll UM (2001) Oncogenes induce and activate endogenous p73 protein. *J Biol Chem* **276**: 11310–11316

Zaika AI, Kovalev S, Marchenko ND and Moll UM (1999) Overexpression of the wild type p73 gene in breast cancer tissues and cell lines. *Cancer Res* **59**: 3257–3263

Zhang SY, Klein-Szanto AJ, Sauter ER, Shafarenko M, Mitsunaga S, Nobori T, Carson DA, Ridge JA and Goodrow TL (1994) Higher frequency of alterations in the p16/CDKN2 gene in squamous cell carcinoma cell lines than in primary tumors of the head and neck. *Cancer Res* **53**: 5050–5053

*© 2001 Cancer Research Campaign*
